# Editorial: Information Processing in the Cerebellum

**DOI:** 10.3389/fnsys.2021.752719

**Published:** 2021-09-17

**Authors:** Conor Houghton, Philippe Isope, Richard Apps, Nadia L. Cerminara

**Affiliations:** ^1^Department of Computer Science, University of Bristol, Bristol, United Kingdom; ^2^Institut des Neurosciences Cellulaires et Intégratives, CNRS, Université de Strasbourg, Strasbourg, France; ^3^School of Physiology, Pharmocology and Neuroscience, University of Bristol, Bristol, United Kingdom

**Keywords:** cerebellum, computational modeling, forward model, timing, complex spike, oscillations, cerebellar learning, cerebellar circuitry

The cerebellum is involved in a wide range of behaviors including the coordination of reflex and voluntary movements, postural adjustments to maintain balance, and the learning of new motor skills. This is also increasing evidence that its role extends to cognition, affect and the control of the autonomic system. Amazingly, the cerebellum contains more than four-fifths of the brain's neurons (Herculano-Houzel, 2009) and so it has an incredible amount of computing power. Considering the cerebellum from a computational perspective has a long history going back to Albus (1971), Marr and Thach (1991), and Ito (1984) and the goal of linking theory to experiment through detailed modeling dates back to the early nineties (Tyrrell and Willshaw, 1992; De Schutter and Bower, 1994a,b). Now, new insights into the anatomy and physiology of cerebellar circuits are leading to revised thinking about how these circuits may process information: multiple sites of plasticity; heterogeneity in structure and function such as zebrin bands; variable complex spike wave form; synchronous activity.

It seems possible that the cerebellum is specialized to perform some specific types of computation. However, it is not known precisely what these might be and there are many, often overlapping, ideas: perceptron learning (Albus, 1971; Ito, 1984; Marr and Thach, 1991), Kalman filtering (Paulin, 1989; Tanaka et al., 2019), forward models (Miall and Wolpert, 1996), expansion coding (Billings et al., 2014), the approximation of cortical feedback (Pemberton et al., 2020), and computing with uncertainty (Palacios et al., 2021). Moreover, differences in cerebellar function are likely to be reflected within regional variations in cerebellar cytoarchitecture (for a review see Cerminara et al., 2015), that in turn reflect different computational roles. This is an important area for studying models of computation: models of cerebellar computation could be effective in describing not just the role of the cerebellum but could inspire universal theories of whole brain function. With this ambition in mind, the scope of this Research Topic was to bring together researchers to showcase recent experimental and computational studies exploring the computational dynamics of the cerebellum at levels from ions and microcircuits.

A fruitful approach to the cerebellum is to describe it using language and ideas borrowed from engineering control systems. Tanaka et al. reviews one such approach. A challenge in designing a control system is that the motor controls need to be based on a current estimate of position even though that estimate may not be available straight away because of the latency in sensory processing. A potential solution is to use a *forward model* (Miall and Wolpert, 1996) to predict the current position from the available, past, sensory data. This review describes evidence, computational and experimental, that cerebellar computation is a forward model. Holland et al. and Anderson et al. consider cerebellar control for the specific example of image stabilization. Holland et al. presents a detailed forward model and compares their simulation to experimental data from mice. In a *filter* inputs are amplified or attenuated as part of processing and Anderson et al. has a cerebellar model of filter learning for an input, in this case the head velocity which eye movements need to compensate.

The exquisite organization of the cerebellar microcircuits and molecular architecture required to support computation has been well-described, however the mechanisms that guide developmental patterning of their afferent inputs is less well-understood. Lackey and Sillitoe show that the effector molecules ephrin-A2/A5 are needed for the parasagittal patterning of spinocerebellar mossy fibers but not the Purkinje cell zonal patterns themselves. Mossy fibers originate from a wide variety of locations in the brain and spinal cord and provide the cerebellum with a rich array of sensory and motor signals converging onto single granule cells (Shimuta et al., 2020). Whether ephrin-A2/A5 is specific to subsets of spinocerebellar mossy fibers or whether this mechanism is generalized across all types of mossy fiber input needs further exploration.

Many proposed mechanisms underlying cerebellar computations require perfectly controlled time processing to avoid any mismatch in comparisons, such as the comparison of predictions with sensory feedback. The cerebellar cortex integrates mossy fiber inputs at a very high frequency so synaptic short-term plasticity properties are important. Notably, Schmidt reviews recent reinvestigations of release properties underlying short-term plasticity at the parallel fiber to Purkinje cell synapse. He develops new hypotheses that can explain how these, the most numerous synapses in the brain, reliably handle inputs at very high frequency. Another critical issue is to understand how local oscillatory activity in cerebellar microcircuits influences incoming information channeling across the cerebellar cortex. Levesque et al. recorded both individual neurons and local field potential (LFP) in the granule cell layer (GCL) of the rat and shows that input driven oscillations in the GCL predict the timing of individual neurons suggesting that LFP may have a preparatory role for cerebellar computation. How this computation is communicated to the rest of the brain remains uncertain. Using dual recordings in the cerebellum and the prefrontal cortex, Tremblay et al. demonstrate that cerebellar stimulation can influence LFP in the prefrontal cortex and that frequency-dependent changes in stimulation drive synchronization of cerebello-cortical and cortico-cortical networks.

Central to cerebellar function are the climbing fibers and the complex spikes they induce in Purkinje cell: cerebellar learning theories suggest climbing fibers signal an error signal to drive depression of the parallel fiber synapses. However, plasticity can occur at multiple sites within the cerebellum and without the need for complex spikes, calling into question the role of climbing fibers in cerebellar learning. Additionally, multiple studies have shown that complex spikes are inherent variability (Najafi et al., 2014; Yang and Lisberger, 2014; Burroughs et al., 2017; Tang et al., 2017). The review paper of Zang and De Schutter highlights recent experimental and computational studies on how this analog complex spike error signal could integrate with cerebellar learning theories. Along similar lines, Yarden-Rabinowitz and Yarom present evidence from previous classical conditioning studies as well as original data that rather than encode an error signal, complex spikes encode the timing of movement initiation through the modification of cerebellar circuitry. Together, these two papers present new views on cerebellar learning based on the graded role of complex spikes.

Together these studies add weight to the notion that the cerebellum has a multiplicity of information processing capabilities. They also demonstrate a wide-diversity of novel insight into the nature and purpose of cerebellar computation, bringing us to the surprising realization that there is no dominant theory of cerebellar function; whether the range of cerebellar operations can be captured by a universal computational algorithm remains a question for the field.

## Author Contributions

All authors contributed to the drafting and editing of this editorial.

## Funding

NC and RA were supported by the Medical Research Council UK (G1100626) and BBSRC (BB/P000959/1). PI was supported by the Centre National pour la Recherche Scientifique (CNRS), the Université de Strasbourg and the Agence Nationale pour la Recherche (ANR-2019-MultiMod, ANR-2019-NetOnTime).

## Conflict of Interest

The authors declare that the research was conducted in the absence of any commercial or financial relationships that could be construed as a potential conflict of interest.

## Publisher's Note

All claims expressed in this article are solely those of the authors and do not necessarily represent those of their affiliated organizations, or those of the publisher, the editors and the reviewers. Any product that may be evaluated in this article, or claim that may be made by its manufacturer, is not guaranteed or endorsed by the publisher.
